# Determination of Ochratoxin A and Ochratoxin B in Archived Tokaj Wines (Vintage 1959–2017) Using On-Line Solid Phase Extraction Coupled to Liquid Chromatography

**DOI:** 10.3390/toxins12120739

**Published:** 2020-11-24

**Authors:** Aneta Kholová, Ivona Lhotská, Adéla Uhrová, Ivan Špánik, Andrea Machyňáková, Petr Solich, František Švec, Dalibor Šatínský

**Affiliations:** 1 The Department of Analytical Chemistry, Faculty of Pharmacy in Hradec Králové, Charles University, Ak. Heyrovského 1203, 500 05 Hradec Králové, Czech Republic; kholovaa@faf.cuni.cz (A.K.); UHROVAAD@faf.cuni.cz (A.U.); solich@faf.cuni.cz (P.S.); svecfr@faf.cuni.cz (F.Š.); satinsky@faf.cuni.cz (D.Š.); 2 The Institute of Analytical Chemistry, Faculty of Chemical and Food Technology, Slovak University of Technology in Bratislava, Radlinského 9, 812 37 Bratislava, Slovakia; ivan.spanik@stuba.sk (I.Š.); andrea.spevak@stuba.sk (A.M.)

**Keywords:** ochratoxin A, ochratoxin B, mycotoxin, Tokaj wine, food control, chromatography, online extraction, column switching

## Abstract

According to the EU legislation, ochratoxin A contamination is controlled in wines. Tokaj wine is a special type of sweet wine produced from botrytized grapes infected by “noble rot” *Botrytis cinerea*. Although a high contamination was reported in sweet wines and noble rot grapes could be susceptible to coinfection with other fungi, including ochratoxigenic species, no screening of Tokaj wines for mycotoxin contamination has been carried out so far. Therefore, we developed an analytical method for the determination of ochratoxin A (OTA) and ochratoxin B (OTB) involving online SPE coupled to HPLC-FD using column switching to achieve the fast and sensitive control of mycotoxin contamination. The method was validated with recoveries ranging from 91.6% to 99.1% with an RSD less than 2%. The limits of quantification were 0.1 and 0.2 µg L^−1^ for OTA and OTB, respectively. The total analysis time of the online SPE-HPLC-FD method was a mere 6 min. This high throughput enables routine analysis. Finally, we carried out an extensive investigation of the ochratoxin contamination in 59 Slovak Tokaj wines of 1959–2017 vintage. Only a few positives were detected. The OTA content in most of the checked wines did not exceed the EU maximum tolerable limit of 2 µg L^−1^, indicating a good quality of winegrowing and storing.

## 1. Introduction

Ochratoxins belong to the group of mycotoxins that are secondary metabolites produced by fungal species growing on plants and plant products with toxic effects on humans and animals [1]. Ochratoxin A (OTA) is the most common and widespread ochratoxin found in a wide variety of agricultural commodities worldwide, ranging from cereal grains to dried fruits and spices to wine, coffee, and cocoa [1,2]. Ochratoxin A is classified as a possible human carcinogen (Group 2B) by the International Agency for Research on Cancer [3]. The most significant negative health impacts of ochratoxin include nephrotoxicity, carcinogenicity, teratogenicity, immunotoxicity, and possibly neurotoxicity [4,5,6]. Naturally occurring ochratoxins are produced by several fungi species, mostly *Aspergillus ochraceus*, *A. carbonarius*, *A. niger*, and *Penicillium verrucosum*. The less common ochratoxin B (OTB), a non-chlorinated OTA analog, is less toxic than OTA [7,8]. However, OTB contamination exceeding OTA levels was found in several red wines from Italy and Spain [9,10]. The European Food Safety Authority set the tolerable weakly intake of OTA at 120 ng per kg body weight, and the European Commission established the maximum permitted OTA content in wine at 2 µg kg^−1^ [11].

Tokaj wine is a special type of sweet wine grown only in the Tokaj wine region at the border of Hungary and Slovakia. The production of Tokaj wine depends on the growth of a necrotrophic fruit fungus *Botrytis cinerea*, called noble rot. The noble rot promotes the dehydration of berries, increasing the sugar concentration. The botrytized grapes make Tokaj wines sweet and full-bodied. However, the distortion of the berries surface by one fungus can favor the berries colonization by others—e.g., pathogenic black *Aspergilli*. The presence of *Aspergillus* and *Penicillium* fungi as potential producers of mycotoxins on botrytized berries in the Tokaj wine region has already been confirmed [12,13]. In addition, Gil-Sena et al. reported the more frequent OTA contamination of sweet wines [14]. According to our best knowledge, no screening of Tokaj wines for ochratoxins has been carried out despite the increased risk so far. 

The most common method for the simultaneous determination of ochratoxin A and B is high performance liquid chromatography (HPLC) coupled with fluorescence detection (FD) [15,16,17,18,19,20,21] or mass spectrometry [22,23,24]. Besides common ELISA methods with spectrophotometric detection, immunosensors have recently become the subject of interest [25,26]. The largest extent of published works involves immunoaffinity columns [15,16,27] for sample preparation, often used in food control laboratories and routine practice. Immunoaffinity-based solid phase extraction (SPE) off-line coupled to HPLC-FD has been adopted as standard by authorities, particularly official method AOAC 2001.01 for wine and beer [28]. Other approaches for ochratoxin extraction from food and drinks are liquid–liquid extraction (LLE) [16], dispersive liquid–liquid microextraction [18], solid-phase microextraction (SPME) [19], solid bar microextraction [20], and magnetic solid-phase extraction [21]. Due to the demanding and lengthy sample preparation prior to chromatographic analysis using immunoaffinity columns, SPE, LLE, and automation or online extraction were also studied. 

Online SPE is an advanced technique combining extraction and subsequent chromatography separation. Coupling these two individual steps in a single analytical procedure can minimize possible human errors and accelerate the entire analysis. This type of extraction relies on the concept of column switching. In the first step, the sample is purified in an extraction cartridge, and the chromatographic separation follows during the next step. Reports dealing with the online extraction and chromatography determination of ochratoxin A in beer [29,30] and wine [30,31,32] have recently been published, but none have focused on OTB. 

Our communication describes the development of a new method for the simultaneous determination of OTA and OTB in Tokaj wines using online SPE-HPLC. A screening study for ochratoxin contamination in botrytized wines was carried out with a set of 59 archived Tokaj wines from the Slovak Tokaj region produced within the period of 1959 to 2017.

## 2. Results

### 2.1. Optimization of the Online SPE-HPLC Procedure

The main goal was to achieve online extraction coupled with chromatographic separation with the efficient removal of matrix interferences and subsequent separation. We focused on the optimization of a method that enables the fast extraction of mycotoxins from the wine with a high recovery.

**Figure 1:**
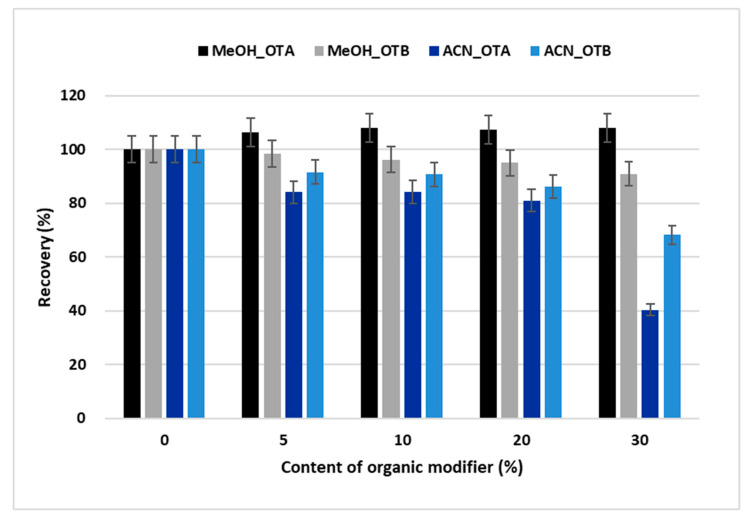
Effect of the percentage of organic solvent in the washing mobile phase on the recovery of ochratoxins A and B after the clean-up step using the online extraction-separation system. The peak area of the pure aqueous washing phase in the absence of organic solvent was set to 100%, and the recoveries of ochratoxins with acetonitrile or methanol were calculated.

**Figure 2:**
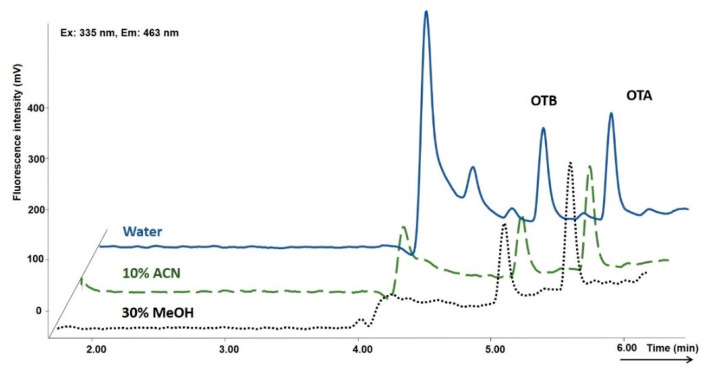
Effect of methanol and acetonitrile content (%) in the washing mobile phase on the clean-up efficiency during the extraction procedure compared to washing with solely water. Time of washing 2 min, chromatographic conditions are reported in Section 5.4.

The ochratoxins A and B are weak organic acids; their formulas are presented in Figure S1. The pKa values for OTA are 3.1 for the carboxylic acid group, 7.9 for the hydroxyl group [33], and 3.3 for the carboxyl group, while the pKa value for the hydroxyl group of OTB is 7.0 [34]. Therefore, all the mobile phases were acidified with a 0.5% acetic acid solution to pH 2.8 to avoid ochratoxin ionization and increase their retention. The optimization of extraction was focused on the fast removal of all interferences while retaining the target compounds. First, the aqueous washing mobile phases with increasing percentages of acetonitrile or methanol were tested. The recovery of both ochratoxins in the absence of organic solvent was set as 100% because water has the lowest elution strength in a reversed phase system comparing to organic solvents, so the least loss of analytes is expected. Acetonitrile is a much stronger eluent. Hence, the partial loss of OTB for the 1 min washing step was observed even when using a 5% aqueous solution. The effect of the washing mobile phase composition on the ochratoxin recovery is presented in Figure 1. Finally, 30% methanol appeared to be a good compromise between the sufficient recovery of both ochratoxins and the ability to remove an interfering matrix. A comparison of chromatograms using pure water, 30% aqueous methanol, and 10% aqueous acetonitrile in the washing mobile phase is demonstrated in Figure 2 under the optimized chromatographic conditions (Section 5.4).

Further optimization concerned the time at which the flow was switched from extraction to analytical column. Efficient clean-up at a flow rate of 2 mL min^−1^ was achieved already in 2 min. Longer washing times did not improve the clean-up efficiency.

Five different stationary phases for the separation of ochratoxins from the matrix interferences were tested: YMC-Triart C18 ExRS, Kinetex Biphenyl, Ascentis Express F5, Ascentis Express RP-Amide, and Kinetex Phenyl-Hexyl. All the columns had the same length of 100 mm and 4.6 mm i.d., while the packing particle sizes ranged from 2.7 to 5 µm. The most efficient separation was achieved using the Kinetex Phenyl-Hexyl stationary phase. Other columns provided a poor selectivity in terms of OTA or OTB coelution with interfering peaks from the matrix while using an online extraction system.

**Figure 3:**
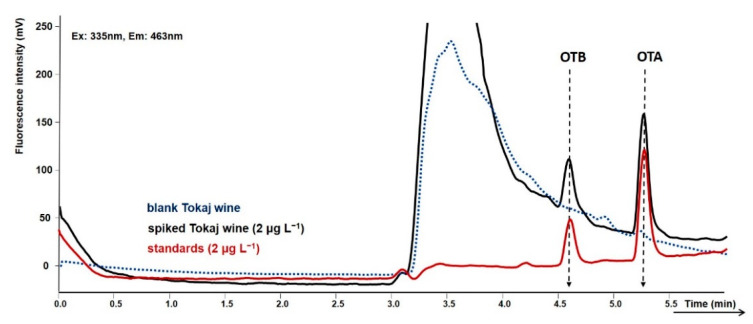
Chromatogram presenting the clean-up and selectivity of the validated method for ochratoxin A and ochratoxin B determination exemplifies with blank Tokaj wine, Tokaj wine spiked with OTA and OTB at a concentration 2 µg L^−1^, and standard at the same concentration. Extraction: Ascentis Express C18 guard column, washing mobile phase: 30% MeOH in 0.5% aqueous acetic acid; time of washing 2 min. Separation: Kinetex Phenyl-Hexyl analytical column; mobile phase: acetonitrile and 0.5% aqueous acetic acid, gradient elution.

**Figure 4:**
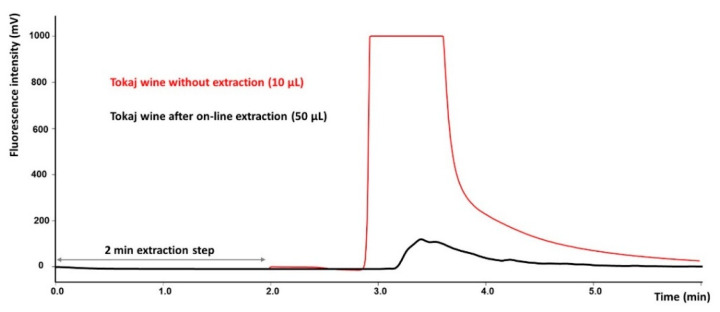
Chromatogram presenting the clean-up efficiency of the on-line SPE-HPLC method. Overloading of the column is observed even with a 10 μL direct injection (no SPE step) in comparison with 50 μL of Tokaj wine loaded on the extraction column and mass reduction during the on-line SPE step. Extraction: Ascentis Express C18 guard column, washing mobile phase: 30% MeOH in 0.5% aqueous acetic acid; time of washing 2 min. Separation: Kinetex Phenyl-Hexyl analytical column; mobile phase: acetonitrile and 0.5% aqueous acetic acid, gradient elution.

The mobile phases for the gradient elution comprised acetonitrile and 0.5% aqueous acetic acid. The acidified mobile phase was used to enhance the retention of ochratoxins in the reversed phase column and to achieve their better separation from polar and weakly retained interferences. The gradient started at 45% acetonitrile, which was maintained for 1 min. The elution of the polar matrix observed at the beginning of the chromatogram was achieved using this mobile phase with a lower elution strength. Then, the acetonitrile percentage was linearly increased to 75% to achieve the complete elution of both ochratoxins from the extraction column and the subsequent separation in the analytical column. The removal of matrix interferences and the separation of both ochratoxins in spiked Tokaj wine is demonstrated in Figure 3. The clean-up efficiency compared to the chromatogram without a separation step is illustrated in Figure 4.

### 2.2. Validation

The method validation concerned the linearity for standard and matrix solutions, repeatability, limits of detection (LOD), limits of quantification (LOQ), intraday precision, and accuracy expressed as recovery. The method validation procedure included the HPLC system suitability test (SST). The results are summarized in Table 1 and Table 2.

Our SST included the repeatability of retention times, peak areas, resolution factor, peak symmetry, and peak capacity to demonstrate the column separation performance after the SPE step and the gradient elution conditions. The repeatability with an RSD of less than 1.21% was calculated from six injections of the standard mixture and spiked matrix solutions at three different concentration levels of 2, 10, and 50 µg L^−1^. Calibration curves were drawn using the measurement of standard solutions and spiked matrix solutions prepared from blank Tokaj wine containing no ochratoxins. The wine was spiked at nine concentration levels, and each sample was injected in triplicate. The linear relationship between the peak area and the ochratoxins concentration was confirmed in the range of 0.2–75 µg L^−1^ for OTB and 0.1–75 µg L^−1^ for OTA. The calibration plots are attached in the Supplementary Materials, Figures S2 and S3. The lowest concentration levels of the calibration range, 0.2 and 0.1 µg L^−1^, were set as the limit of quantification for OTB and OTA, respectively. The limit of detection was calculated from the relationship between LOQ and LOD to the signal to noise ratio, as 3/10 of LOQ. Moreover, the LOQ values were confirmed experimentally by the multiple dilution of standard solutions. The precision and recovery of the proposed method, including an online extraction step, were evaluated by the repetitive determination of six spiked wine samples at a concentration level of 2 µg L^−1^ for both ochratoxins. This concentration corresponded to the maximum levels for OTA in wine permitted by the European Union regulation. The RSD of the method did not exceed 2%. The method accuracy was calculated as the recovery (peak area of the spiked sample with standard solution divided by the peak area of the standard solution at the same concentration level) × 100%. The calculated mean recoveries were 91.7% and 99.1% for OTA and OTB, respectively. The method recovery of 93–103% was confirmed at two more concentration levels, 10 and 50 µg L^−1^, by the comparison of the peak areas of the spiked matrix and standard solution.

### 2.3. Tokaj Wine Analysis

The Tokaj wine is undoubtedly a more complex matrix than a standard dry white wine due to a load of sugars and its origin in botrytized berries. Therefore, a novel fully automated method was optimized, dealing with the determination of two naturally occurring ochratoxins in a specific Tokaj wine matrix. A set of 59 archived Tokaj wines was obtained from Slovak vineyards. Different varieties, wine products varying in sugar and alcohol content, and vintages were included in the set. Each wine (50 µL) was directly injected in the chromatographic system for analysis. The levels found in contaminated wines are summarized in Table 3. 

**Figure 5:**
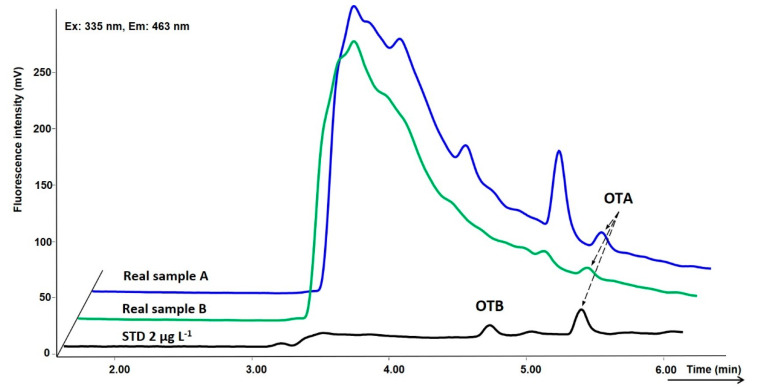
Representative chromatogram of Tokaj wine samples in comparison to the standard at the conc. level 2 µg L^−1^. Sample A: Archívné víno 6-Put, Zlatý Strapec (1993) (OTA 1.2 µg L^−1^); Sample B: Archívné víno Esencia, Ostrožovič (2000) (OTA 0.8 µg L^−1^). For conditions, see Figure 3.

The list of all tested wines with contamination levels lower than LOQ is presented in the Supplementary Materials Table S1. The OTA and OTB contents in the wines were far below the permitted maximum limits of 2 µg L^−1^ for OTA. The highest OTA concentration of 1.2 µg L^−1^ was found in one wine, and only four wines turned out to be positive. Representative online SPE chromatograms of two contaminated Tokaj wines are presented in Figure 5.

## 3. Discussion

The proposed method enables the fast determination of two ochratoxins in Tokaj wines. Unlike the Official AOAC method using immunoaffinity clean-up, we achieved the full automation of the extraction process coupled to chromatographic analysis with the same limit of quantification for OTA, 0.1 μg L^−1^. The on-line extraction approach enables a high sample throughput and the elimination of human errors and laborious manual handling. Precise timing, programming, flow rate control, and direct elution to chromatographic column provide a good repeatability. The on-line approach leads to green chemistry and savings due to the elimination of disposable SPE cartridges. 

The recovery of both ochratoxins is improved in comparison with previous on-line SPE methods for the determination of OTA in wine, 92% in our method vs. 82% reported by Campone et al. [32] and 76% documented by Bacaloni et al. [30]. Although Armutcu et al. [31] showed a good recovery of 105%, their method lacked our sensitivity, with a LOQ 0.5 μg L^−1^. None of the method challenged the determination of the more polar ochratoxin, OTB, that is difficult to separate from the wine matrix. Moreover, our novel method is notably faster, with only 6 min including the on-line SPE step, which distinguishes it from the 22–35 min methods using online extractions in wine [30,31,32]. 

Regarding the screening results of archived Tokaj wines, only a few samples were found to be positive in contrast to our expectations. However, any objections to OTA stability during archiving can be refuted. Ochratoxin A is a very persistent mycotoxin and is not considered to be reduced during wine aging. Several studies have dealt with its reduction throughout the winemaking process using solid mass adsorption and fermentation or by an additional laborious process [35,36]. Terminating any processes before storing, OTA is stable in an acidic medium. No carbohydrate adducts were observed unless treated with a high temperature [37]. On the contrary, the hydrolysis of ochratoxin C to ochratoxin A, resulting in an increase in the OTA levels with time, was described by Remiro [38]. 

We did not confirm the contamination caused by *Aspergillus* and *Penicillium* fungi as potential producers of mycotoxins on botrytized berries demonstrated in Bene’s and Mayar’s report [12]. A possible reason for the low levels of ochratoxins could be the massive growth of the noble mold that protects the grapes from the impact of other toxigenic mold species.

## 4. Conclusions

We present, for the first time, a report dealing with the potential contamination of Tokaj wines with two naturally occurring ochratoxins. Our approach to the simultaneous extraction and determination of ochratoxin A and ochratoxin B in wine is based on using a fused core particle sorbent for SPE coupled with HPLC-FD. The developed matrix clean-up step was automated, rapid, simple, and had a high sample throughput. The results of the method validation demonstrated a good linearity, precision, sensitivity, recovery, and reproducibility. The online SPE procedure with the relatively large volume injection demonstrates that our technique is a method of choice for the sensitive, efficient, and rapid analysis of trace levels of mycotoxins in wines. The limits of quantification are ten- to twenty-fold lower than the maximum level of OTA permitted in wines. The EU OTA limit was not exceeded in any of the tested wine. Ochratoxin B has no defined limit for the contamination of wine, and its content did not exceed the LOQ. Our results indicate the good quality of the winegrowing and storing of Slovak Tokaj wines.

## 5. Materials and Methods

### 5.1. Reagents and Materials

Standards of ochratoxin A (≥98%), ochratoxin B (10 µg mL^−1^ in acetonitrile; ≥98%), glacial acetic acid, and organic solvents (HPLC gradient grade) methanol and acetonitrile were obtained from Sigma-Aldrich (Prague, Czech Republic). The ultra-pure water used for the preparation of the mobile phase was produced in a Milli-Q system (Millipore, Bedford, MA, USA). Other chemicals and used materials were of analytical grade. Five different analytical columns were tested: YMC-Triart C18 ExRS (100 × 4.6 mm, particle size 3 µm) (YMC, Kyoto, Japan), Kinetex Biphenyl (100 × 4.6 mm, particle size 5 µm) (Phenomenex, Torrance, USA), Ascentis Express F5 (100 × 4.6 mm, particle size 5 µm) (Sigma Aldrich, Bellefonte, USA), Ascentis Express RP-Amide (100 × 4.6 mm, particle size 2.7 µm) (Sigma Aldrich), and Kinetex Phenyl-Hexyl (100 × 4.6 mm, particle size 2.6 µm) (Phenomenex). Guard column Ascentis Express C18 (5 × 4.6 mm, particle size 2.7 µm) (Sigma Aldrich) was applied for ochratoxin extraction and sample purification.

The 59 archival Tokaj wines with 3–6 puttonyos (unit given to denote the level of sugar and hence the sweetness) of vintages 1959–2017 originated from Slovak Tokaj region vineyards Zlatý Strapec in Viničky as well as from the vineyards of the Ostrožovič company, and Tokaj & CO in Malá Trňa. The oldest Tokaj wine from 1959 that had 5-puttonyos was from vineyard Zlatý Strapec. The complete list of all the samples with their characterization is summarized in Table S1. The alcohol content of Tokaj wines is usually 12–14%, with the exception of essences characterized by very intense sweetness but a low alcohol content about 5–7% [39]. In advance, dry and sweet wines from the same vineyards but without fortification with botrytized berries puttonyos were included in the sample selection.

The collection of all wines, 8 mL from each sample in a dark vial, was kept at 4 °C before the chromatography analysis. Blank Tokaj wine not containing ochratoxins used for the optimization and validation of the method was purchased in a local supermarket and spiked with a standard solution. The absence of contamination by OTA and OTB below the LOD or the absence of any other interference eluted in the same retention time was confirmed by chromatographic analysis (see Figure 3).

### 5.2. Instrumentation and Software

The chromatography system Shimadzu Prominence (Shimadzu Corporation, Kyoto, Japan) was used for the method development and validation. The system consisted of a dual-pump module LC-20 AD, a DGU-AS mobile phase degasser, an autosampler SIL-20 AC, a CTO-20AC column oven with an FCV-12AH high-pressure six-port switching valve, and a fluorescence detector RF-10A XL. The HPLC system was controlled by a CBM-20A communication module. Lab-Solution software (Shimadzu Corporation) was used for the data acquisition and evaluation.

### 5.3. Preparation of Standards Solutions

A standard stock solution of OTB at a concentration of 100 µg L^−1^ was prepared by diluting the original standard solution in 0.5% aqueous acetic acid. A standard solution of OTA at a concentration of 100 µg L^−1^ was obtained by dissolving in methanol. Both solutions were stored at −20 °C. Working solutions for the optimization and validation of the method were obtained by diluting the standard stock solution in 0.5% aqueous acetic acid solution to obtain solutions with concentrations in a range of 0.2–75 µg L^−1^. Working solutions for matrix calibration were prepared in the same way and the same concentration range by dilution with blank Tokaj wine. All the solutions were stored at 4 °C in the dark. Each untreated and undiluted wine (50 µL) was injected directly into the online SPE-HPLC system. All the samples and working standard solutions were prepared fresh daily and injected in triplicate.

### 5.4. HPLC Column-Switching Analysis

Online extraction and the simultaneous determination of OTA and OTB were carried out using a column switching HPLC system (Figure S4). The concept of the column switching relies on the connection of an extraction column with an analytical column via a six-port switching valve. A 50 µL sample was injected and loaded in the extraction cartridge at the first valve position and cleaned using the washing mobile phase for 2 min. A fused-core Ascentis Express RP C18 (5 × 4.6 mm, particle size 2.7 µm) extraction cartridge was used for the ochratoxin extraction. Washing with the mobile phase consisting of methanol acidified with 0.5% aqueous acetic acid to pH 2.8 (30:70, *v*/*v*) was carried out at a flow rate of 2.0 mL min^−1^ and temperature of 50 °C to remove matrix interferences. Polar interferences from the matrix were removed at this step, while the analytes were preconcentrated in the cartridge. After 2 min, the valve was switched and ochratoxins were eluted from the extraction cartridge to the Kinetex Phenyl-Hexyl (100 × 4.6 mm, particle size 2.6 µm) analytical column and separated via gradient elution. The gradient elution at a flow rate of 1.0 mL min^−1^ and a column temperature of 50 °C started with an isocratic step of 45% acetonitrile for 1 min, followed by a linear increase to 75% acetonitrile in 90 s. After 30 s of plateau, the organic percentage was decreased back to 45% and the valve was switched back to the loading position. The analytical column was equilibrated to the initial conditions during the next sample pretreatment step. The total analysis time was less than 6 min. The emission and excitation wavelengths of the fluorescence detector—Ex 335 and Em 463 nm—were chosen based on the fluorescence spectra of OTA and OTB.

## Figures and Tables

**Table 1:** Online SPE-HPLC system suitability parameters.

Analyte	Retention Time (min)	Ret. Time RSD (%) ^1^	Peak Capacity ^2^	Peak Symmetry ^3^	Peak Areas Repeatability RSD (%) ^4^	R_S_^5^
OTA	5.27	≤0.1	11.44	1.203	0.84; 0.54; 0.92	4.14
OTB	4.59	≤0.1	11.23	1.211	1.21; 0.48; 1.08	

^1^ Number of replicates, n = 6; ^2^ peak capacity expressing the efficiency of the method (gradient elution) is calculated as P_c_ = (the gradient time/4 × peak width in half) + 1 (times not included online SPE step); ^3^ peak symmetry was calculated by the Lab Solution software (ratio of descending to ascending part of peak in 10% of height); ^4^ RSD was calculated from six injections of standard mixture at concentration levels: c_1_ = 2 µg L^−1^, c_2_ = 10 µg L^−1^, c_3_ = 50 µg L^−1^; ^5^ resolution factor for the separation of OTA from OTB.

**Table 2:** Analytical characteristics of the validated online SPE–HPLC method used for the detection of ochratoxins in Tokaj wines.

Validation Parameters	OTA	OTB
Standard linear calibration range (µg L^−1^) ^1^	0.1–75	0.2–75
Slope	74,443 ± 750	34,818 ± 904
Intercept	−46,480 ± 22,627	−4219 ± 28,743
Regression coefficient (r^2^)	0.9996	0.9977
Matrix (Tokaj wine) linear calibration range (µg L^−1^) ^1^	0.1–75	0.2–75
Slope	73,837 ± 581	32,986 ± 786
Intercept	−20,233 ± 17,547	22,092 ± 25,014
Regression coefficient (r^2^)	0.9998	0.9980
LOD (µg L^−1^)	0.03	0.06
LOQ (µg L^−1^)	0.10	0.20
Precision (RSD, %) ^2^	1.60	0.63
Interday precision (RSD, %) ^3^	6.81	1.79
Accuracy-spike recovery (%) ± SD ^4^	91.69 ± 1.64	99.07 ± 0.58

^1^ Each concentration level was measured in triplicate; ^2^ repetitive determination of six spiked Tokaj wine samples at one concentration level, 2 µg L^−1^; ^3^ interday precision was measured in triplicate in four various days; ^4^ accuracy was determined as a method recovery using six spiked Tokaj wine samples at a concentration level of 2 µg L^−1^, each in triplicate (± the minimal and maximal standard deviation of recovery determination).

**Table 3:** Content of ochratoxins in Tokaj wines that were found to be contaminated.

OTA (EU limit: 2 µg L^−1^)	Archívné víno 6-Put, Zlatý Strapec 1993	1.22 µg L^−1^
	Archívné víno Esencia, Ostrožovič 2000	0.85 µg L^−1^
	Muškát žltý, Slámové víno, Ostrožovič 2010	0.72 µg L^−1^
	Archívné víno 6-Put, Ostrožovič 1999	0.37 µg L^−1^
OTB	None found	

## Supplementary Materials

The following are available online at https://www.mdpi.com/2072-6651/12/12/739/s1. Figure S1: Chemical structures of analyzed ochratoxins; Figure S2: Matrix calibration plot for OTA; Figure S3: Matrix calibration plot for OTB; Figure S4: Schematic of online SPE–HPLC system for ochratoxins extraction and determination in Tokaj wines; Table S1: The list of archival Tokaj wines tested for ochratoxin contamination.
