# Supplementary material for: Determination of Ochratoxin A and Ochratoxin B in Archived Tokaj Wines (Vintage 1959–2017) Using On-Line Solid Phase Extraction Coupled to Liquid Chromatography
Source: Toxins (Basel). 2020 Nov 24;12(12):739. doi: 10.3390/toxins12120739 (PMC7761308; doi:10.3390/toxins12120739)
Supplement: Supplementary file 1 [file toxins-12-00739-s001.pdf]

Toxins **2020**, *12*, 739; doi:10.3390/toxins12120739

Supplementary Materials: Determination of Ochratoxin A and Ochratoxin B in Archived Tokaj Wines (Vintage 1959–2017) Using On-Line Solid Phase Extraction Coupled to Liquid Chromatography

Aneta Kholová, Ivona Lhotská, Adéla Uhrová, Ivan Špánik, Andrea Machyňáková, Petr Solich, František Švec and Dalibor Šatínský

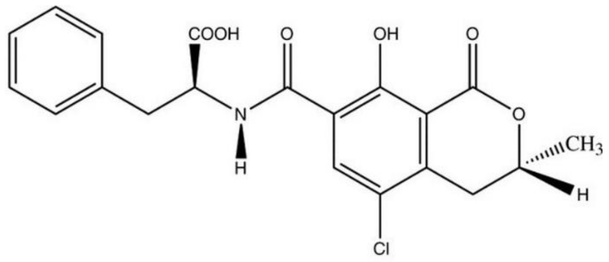

Ochratoxin A

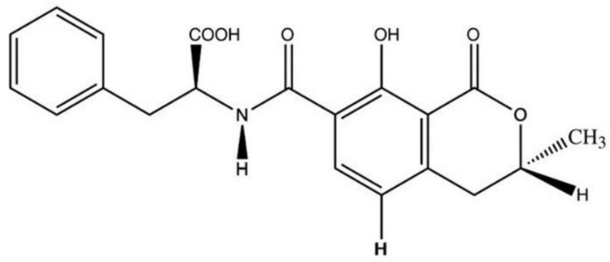

Ochratoxin B

Figure S1. Chemical structures of analyzed ochratoxins .

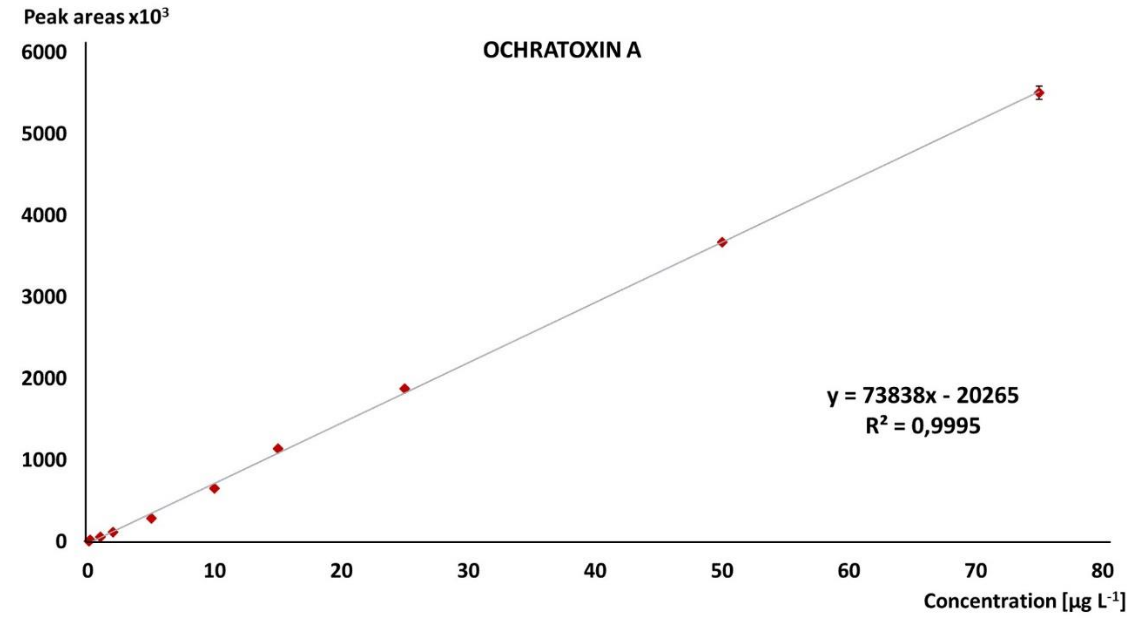

Figure S2. Matrix calibration plot for OTA .

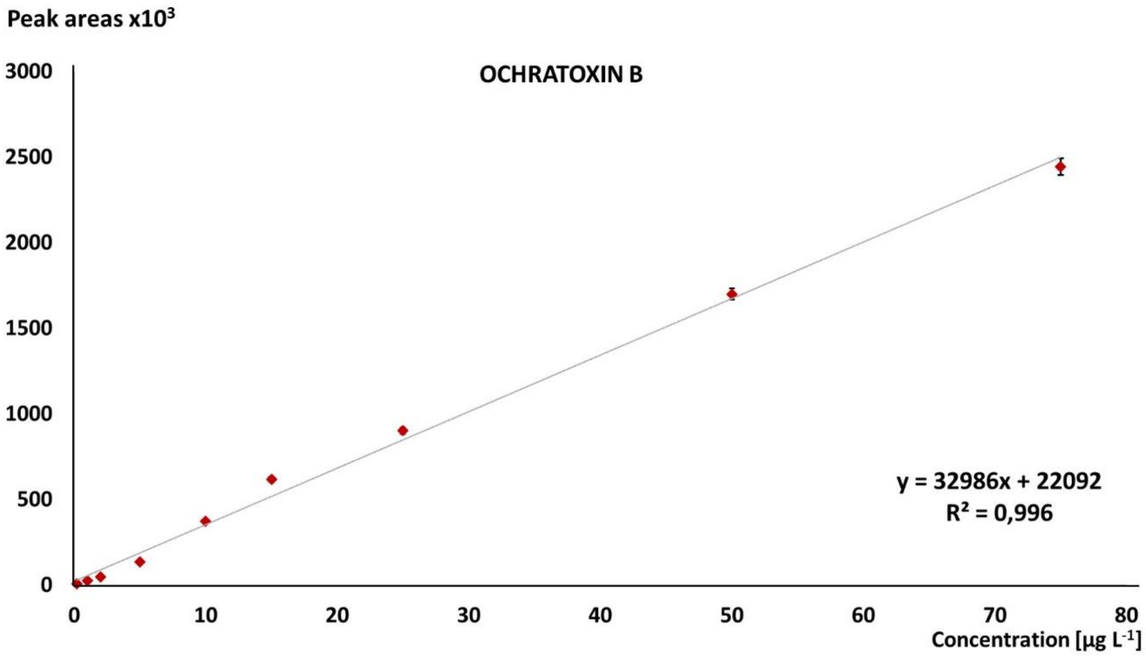

Figure S3. Matrix calibration plot for OTB.

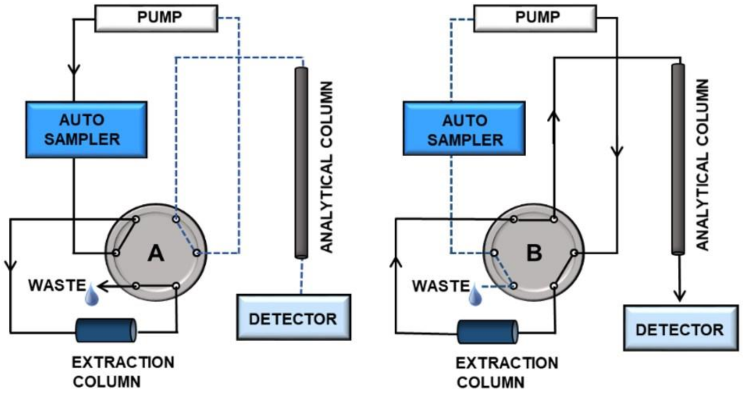

Figure S4. Schematic of online SPE–HPLC system for ochratoxins extraction and determination in Tokaj wines. Valve position A—extraction and ochratoxins preconcentration; valve position B—separation of ochratoxins in analytical column.

Table S1. The list of archival Tokaj wines tested for ochratoxins contamination.

		Puttony	Vintage	Winery	c OTB (µg L^{-1})	c OTA (µg L^{-1})
			Essence—nectar from botrytized berries [1]			
1	Archívné víno Esencia	> 6	1999	Tokaj&Co	< LOD	< LOD
2	Archívné víno Esencia	> 6	2000	Ostrožovič	< LOD	0.85
3	Tokajská Esencia	> 6	2009	Tokaj&Co	< LOD	< LOD
			Aszu wine—wine fortified with the puttonyos of botrytized berries [2]			
4	Tokajské archívné víno	5	1959	Zlatý Strapec	< LOD	< LOD
5	Tokajské archívné víno	6	1972	Zlatý Strapec	< LOD	< LOD
6	Tokajské archívné víno	5	1972	Zlatý Strapec	< LOD	< LOD
7	Archívné víno	5	1983	Zlatý Strapec	< LOD	< LOQ
8	Tokajské archívné víno	6	1983		< LOD	< LOD
9	Archívné víno	3	1988	Zlatý Strapec	< LOD	< LOD
10	Archívné víno	5	1989	Ostrožovič	< LOD	< LOD
11	Archívné víno	6	1989	Ostrožovič	< LOD	< LOD
12	Archívné víno	6	1989	Tokaj&Co	< LOD	< LOD
13	Archívné víno	2	1989	Tokaj&Co	< LOD	< LOQ
14	Archívné víno	2	1990	Tokaj&Co	< LOD	< LOD
15	Archívné víno	5	1990	Tokaj&Co	< LOD	< LOD
16	Archívné víno	3	1990	Tokaj&Co	< LOD	< LOD
17	Archívné víno	6	1993	Zlatý Strapec	< LOD	1.22
18	Archívné víno	5	1993	Zlatý Strapec	< LOD	< LOD
19	Archívné víno	6	1993	Ostrožovič	< LOD	< LOD
20	Tokajský výběr	4	1993	Zlatý Strapec	< LOD	< LOD
21	Archívné víno	6	1993	Tokaj&Co	< LOD	< LOD
22	Archívné víno	5	1993	Ostrožovič	< LOD	< LOD
23	Archívné víno	3	1995	Ostrožovič	< LOD	< LOQ
24	Archívné víno	4	1995	Tokaj&Co	< LOD	< LOD
25	Tokajský výběr	3	1995	Zlatý Strapec	< LOD	< LOD
26	Archívné víno	6	1997	Zlatý Strapec	< LOD	< LOD
27	Archívné víno	4	1999	Ostrožovič	< LOD	< LOD
28	Archívné víno	3	1999	Ostrožovič	< LOD	< LOQ
29	Archívné víno	6	1999	Ostrožovič	< LOD	0.37
30	Tokajský výběr; L1	4	2000	Zlatý Strapec	< LOD	< LOD
31	Tokajský výběr	3	2000	Zlatý Strapec	< LOD	< LOD
32	Tokajský výběr	5	2000	Zlatý Strapec	< LOD	< LOQ
33	Archívné víno	6	2002	Ostrožovič	< LOD	< LOQ

34	Archívné víno	4	2002	Ostrožovič	< LOD	< LOD
35	Archívné víno	3	2003	Ostrožovič	< LOD	< LOD
36	Tokajský výběr	6	2003	Ostrožovič	< LOD	< LOD
37	Archívné víno	5	2003	Ostrožovič	< LOD	< LOD
38	Výběr	5	2003	Tokaj&Co	< LOD	< LOD
39	Archívné víno	5	2004	Ostrožovič	< LOD	< LOQ
40	Archívné víno	4	2004	Ostrožovič	< LOD	< LOD
41	Výběr	6	2006	Tokaj&Co	< LOD	< LOD
42	Výběr	4	2009	Tokaj&Co	< LOD	< LOD
43	Výběr	3	2009	Tokaj&Co	< LOD	< LOD
44	Tokaj	4	2016	Ostrožovič	< LOD	< LOD
			Others [3]			
45	Archívné víno samorodné sladké	–	1997	Tokaj&Co	< LOD	< LOD
46	Samorodné suché	–	1997	Zlatý Strapec	< LOD	< LOD
47	Samorodné sladké	–	2006	Tokaj&Co	< LOD	< LOD
48	Samorodné suché	–	2009	Tokaj&Co	< LOD	< LOD
49	Muškát žltý, Slámové víno	–	2010	Ostrožovič	< LOD	0.72
50	Tokajský Forditáš	–	2011	Tokaj&Co	< LOD	< LOD
51	Muškát žltý, Cibébový výběr	–	2012	Ostrožovič	< LOD	< LOD
52	Furmint, Cibébový výběr	–	2012	Ostrožovič	< LOD	< LOD
53	Furmint, Cibébový výběr	–	2013	Ostrožovič	< LOD	< LOD
54	Furmint	–	2014	Ostrožovič	< LOD	< LOD
55	Muškát žltý	–	2015	Ostrožovič	< LOD	< LOD
55	Lipovina	–	2015	Ostrožovič	< LOD	< LOD
56	Samorodné suché	–	2016	Ostrožovič	< LOD	< LOD
58	Šupkáč-90 dní Furmint	–	2017		< LOD	< LOD
59	Furmint-Šupkáč 30 dní	–	2017		< LOD	< LOQ

[1] Esencia has the highest sugar content with lower alcohol content (5–10%); [2] Number of puttonyos indicates amount of botrytized berries added and thus implies the wine sweetness. The alcohol content can be higher than in ordinary wines (12–14%); [3] It is originated from the same vineyards by various techniques, the amount of botrytized berries is not specified.
